# FGF21 ameliorates diabetic nephropathy through CDK1-dependently regulating the cell cycle

**DOI:** 10.3389/fphar.2024.1500458

**Published:** 2025-01-03

**Authors:** Yudie Zhang, Fan Wang, Chongyang Zhang, Fan Yao, Bin Zhang, Yongping Zhang, Xiaobo Sun

**Affiliations:** ^1^ Guizhou University of Traditional Chinese Medicine, Guiyang, China; ^2^ Institute of Medicinal Plant Development, Peking Union Medical College and Chinese Academy of Medical Sciences, Beijing, China; ^3^ Key Laboratory of Bioactive Substances and Resources Utilization of Chinese Herbal Medicine, Ministry of Education, Beijing, China; ^4^ Beijing Key Laboratory of Innovative Drug Discovery of Traditional Chinese Medicine (Natural Medicine) and Translational Medicine, Beijing, China; ^5^ Key Laboratory of efficacy evaluation of Chinese Medicine Against Glyeolipid Metabolism Disorder Disease, State Administration of Traditional Chinese Medicine, Beijing, China

**Keywords:** FGF21, diabetic nephropathy, CDK1, cell cycle, glomerular filtration barrier

## Abstract

**Background:**

Diabetic nephropathy (DN) is a prevalent global renal illness and one of the main causes of end-stage renal disease (ESRD). FGF21 has been shown to ameliorate diabetic nephropathy, and in addition FGF-21-treated mice impeded mitogenicity, whereas it is unclear whether FGF21 can influence DN progression by regulating the cell cycle in diabetic nephropathy.

**Methods:**

In order to create a diabetic model, STZ injections were given to C57BL/6J mice for this investigation. Then, FGF21 was administered, and renal tissue examination and pathological observation were combined with an assessment of glomerular injury, inflammation, oxidative stress, and the fibrinogen system in mice following the administration of the intervention. Furthermore, we used db/db mice and FGF21 direct therapy for 8 weeks to investigate changes in fasting glucose and creatinine expression as well as pathological changes in glomeruli glycogen deposition, fibrosis, and nephrin expression. To investigate the mechanism of action of FGF21 in the treatment of glycolytic kidney, transcriptome sequencing of renal tissues and KEGG pathway enrichment analysis of differential genes were performed.

**Results:**

The study’s findings demonstrated that FGF21 intervention increased clotting time, decreased oxidative stress and inflammation, and avoided thrombosis in addition to considerably improving glomerular filtration damage. After 8 weeks of FGF21 treatment, glomerular glycogen deposition, fibrosis, and renin expression decreased in db/db mice. Moreover, there was a notable reduction of creatinine and fasting blood glucose levels. Additionally, the CDK1 gene, a key player in controlling the cell cycle, was discovered through examination of the transcriptome sequencing data. It was also shown that FGF21 dramatically reduces the expression of CDK1, which may help diabetic nephropathy by averting mitotic catastrophe and changing the renal cell cycle.

**Conclusion:**

In short, FGF21 improved the development of diabetic nephropathy in diabetic nephropathy-affected animals by reducing glomerular filtration damage, inflammation, and oxidative stress, inhibiting the formation of thrombus, and controlling the cell cycle through CDK1.

## 1 Introduction

Diabetes is becoming more common as people’s lifestyles and material circumstances change. Diabetes affects over 415 million people globally (Falkevall et al., 2017). Diabetes-related microvascular complications such as diabetic nephropathy (DN) are widespread and represent a major global health issue. It is also a major cause of end-stage renal disease (ESRD) with a high incidence rate (Wu et al., 2018). Many investigations have shown that lipid metabolism abnormalities are the primary cause of DN and that they worsen over time. DN is usually defined by changes in metabolism and hemodynamics brought on by hyperglycemia (Jiang et al., 2005; Goldberg et al., 2012; Liu et al., 2009). Metabolic disorders also cause oxidative stress, inflammation, and renal fibrosis (Grabias and Konstantopoulos, 2014; Alicic et al., 2017; Tuttle et al., 1991). A further sign of diabetic nephropathy (DN) is deterioration of the glomerular filtration barrier, which is characterized by damage to glomerular podocytes that develop epithelial mesenchymal transition (EMT) in response to particular stimuli and abnormalities in markers specific to podocytes. At EMT, podocytes separate from the glomerular basement membrane, flush into the urine, and eventually exit the body (Simonson, 2007). The podocyte serves as the last line of defense to stop protein loss from the blood as it passes through the glomerulus. Thus, podocytes play a crucial role in preserving the glomerular filtration barrier (Haley et al., 2018).

Renal fibrosis occurs in the later stages of diabetic nephropathy progression. During this period, endothelial-mesenchymal transition (EndMT) is a common cellular phenotypic transition in which endothelial cells lose specific morphology and acquire myofibroblast-like features including α-smooth muscle actin (αSMA), poikilodulin, and fibronectin. Podocyte epithelial-mesenchymal transition (EMT) is a maladaptive response to injury that results in impaired glomerular filtration barrier integrity and proteinuria (Dong et al., 2023; Manetti et al., 2017). Wnt/β-catenin signaling plays a key role in promoting podocyte injury and dysfunction (Dai et al., 2009). Wnts induces dephosphorylation of β-catenin and stimulates its translocation to the nucleus, whereas β-catenin activation inhibits the expression of renin expression, specifically disrupting the slit septum of the glomerular filtration membrane (Chen et al., 2022; MacDonald et al., 2009). In addition to the above signaling, Notch signaling and Hedgehog signaling play important roles in the development of diabetic nephropathy (Tuttle et al., 2022; Zhao et al., 2023). Notably, it has been shown that endothelial glucocorticoid receptor (GR) deficiency leads to upregulation of the classical Wnt signaling pathway, impaired fatty acid oxidation and thus accelerated fibrosis (Przybyciński et al., 2021; Srivastava et al., 2021a). Interestingly, GR deficiency in podocytes leads to similar results (Srivastava et al., 2021b). Similarly, FGFR1 and SIRT3-mediated mechanisms have been shown to ameliorate diabetic renal fibrosis (Li et al., 2020a; Srivastava et al., 2018). Depending on the pathogenesis of DN, some potential drugs corresponding to DN have also been shown to have therapeutic effects on DN in recent years, such as empagliflozin, where Linagliptin -mediated inhibition of dipeptidyl peptidase 4 (DPP4) hinders EMT to ameliorate renal fibrosis in DN (Kanasaki et al., 2014; Li et al., 2020b). In addition, sodium-glucose cotransporter protein-2 inhibitors, glycolysis inhibitors, mineralocorticoid antagonists, renin-angiotensin system inhibitors, and the peptide AcSDKP ameliorate DN by improving glycolysis, metabolic reprogramming, and inhibiting EMT (Li et al., 2017; Srivastava et al., 2020a; Srivastava et al., 2020b; Shi and Bhalla, 2023;Lin et al., 2024).

FGF21 is a member of the fibroblast growth factor (FGF) superfamily, which is a collection of signaling proteins with 150–300 amino acids with a conserved core of about 120 amino acids. It was initially discovered in 2000. The human chromosome 19 gene FGF21 is 75% homologous to the mouse FGF21 gene (Nishimura et al., 2000; Itoh and Ornitz, 2011). Early-stage diabetic nephropathy is intimately linked to FGF21 (Esteghamati et al., 2017; Xu et al., 2017). It has been demonstrated that little daily doses of FGF21 markedly improved morphological glomerular defects, insulin resistance, and lipid levels in diabetic db/db mice (Kim et al., 2013). Type 1 diabetic nephropathy is lessened by FGF21-mediated activation of the AMPK and antioxidant pathways (Weng et al., 2020; Cheng et al., 2016; Cheng et al., 2020). A deficit in FGF21 exacerbates inflammation, oxidative stress, and fibrosis in free fatty acid-induced nephropathy (Zhang et al., 2013).

Numerous investigations have demonstrated that podocyte loss over a particular threshold results in glomerulosclerosis, suggesting that podocytes are the cause of most glomerular disorders (Kriz and LeHir, 2005; Kim et al., 2001; Kretzler, 2005). A lower number of podocytes is a predictor of diabetic nephropathy because the degree of podocyte reduction influences the degree of proteinuria (Barisoni et al., 2007; Wiggins, 2007). Furthermore, the loss of podocytes is linked to the mitotic catastrophe it is going through (Lasagni et al., 2010; Kriz et al., 1995). Furthermore it was found that premature activation of CDK1 can lead to mitotic catastrophe (Castedo et al., 2002). Importantly, FGF-21-treated mice impeded mitogenicity and inhibited hepatocyte value-addition (Kharitonenkov et al., 2005). While there is a lack of research on whether FGF21 can affect the progression of DN by regulating the cell cycle in diabetic nephropathy, this article is based on this to explore the novel mechanism of FGF21 in the treatment of diabetic nephropathy.

## 2 Material and methods

### 2.1 Reagents

Recombinant FGF21 protein (KYS202002, later referred to as FGF21) was provided by Jiangsu Kangyuan Pharmaceuticals Co., Ltd. (Jiangsu, China). Engeletin was purchased from Master of Bioactive Molecules (USA). Electronic balance was purchased from Mettler Toledo Instruments (Shanghai) Co., Ltd. (Shanghai, China). AU680 automatic biochemical analyzer was purchased from Beckman Coulter (United States).

### 2.2 Animal

Male 5-week-old db/db and dbm mice were purchased from Jiangsu Xishan Animal Technology (Jiangsu, China), and male 7-8-week-old C57BL/6J mice were purchased from Beijing Vitae Laboratory Animal Science and Technology (Beijing, China), and were housed in ventilated cages at temperature (20°C–25°C), relative humidity (60%), light/dark cycle (12 h), and free access to food and water, etc. were maintained in ventilated cages. All animal care and experimental procedures were ethically approved by the Animal Care and Use Committee of the Chinese Academy of Medical Sciences and Peking Union Medical College Affiliated Institutions. Measures were taken to reduce the number of animals used and to ensure that suffering was minimized.

#### 2.2.1 STZ-induced diabetic nephropathy model

Eighty 7–8 weeks old male C57BL/6J mice were taken from the streptozotocin (STZ) diabetic nephropathy model and randomly divided into 6 groups of 12 mice each. One group was a blank control group, which was injected with saline after 4 weeks of normal diet. The remaining group of mice, after 4 weeks of high-fat diet (HFD), were injected once with STZ (140 mg/kg) to induce the diabetic nephropathy model. Three of the groups were FGF21 administration groups, which were injected subcutaneously with FGF21 after modeling, namely FGF21-L [HFD + STZ + FGF21 (3 mg/kg)], FGF21-M [HFD + STZ + FGF21 (6 mg/kg)], and FGF21-H [HFD + STZ + FGF21 (12 mg/kg)]. The last group was the positive drug control group, where mice modeled for gavage administration of engeletin. After these mice were administered for 4 weeks, fasting blood glucose was tested, urine was collected, and blood and kidneys were taken after anesthesia for later experiments.

#### 2.2.2 Spontaneous type II diabetes model in dbdb mice

After a week of acclimation feeding, five-week-old db/db mice were split into three groups of ten mice each: the Model group, the FGF21 treatment group, and the other group. A low dose group [FGF21-L, FGF21 (3 mg/kg)] and a high dose group [FGF21-H FGF21 (6 mg/kg)] were created from the administered groups. Ten additional dbm mice served as the blank control group. After 8 weeks of administering FGF21 subcutaneously in the administered group, in addition to the blank group and model group by saline gavage, fasting blood glucose was assessed, blood was collected for biochemical testing, and kidneys were removed to observe pathological alterations.

### 2.3 Ethics approval

All animal experiments were approved by the Experimental Animal Ethics Committee of the Institute of Medicinal Plant Development, Peking Union Medical College, in accordance with the National Guidelines for the Care and Use of Animals (SCXK-2012-0001, SYXK-2023-0008).

### 2.4 Biochemical assays

#### 2.4.1 Urine tests

Urine was collected from each group after 4 weeks of drug administration, and urine albumin (uALB), albumin (ALB), uric acid (UA) levels, β2-microglobulin (β2-MG) with kidney injury factor-1 (KIM-1) levels and N-acetyl-b-D-glucosaminoglycans (NAG) levels were measured by using automatic biochemistry instrument.

#### 2.4.2 Serum indicator tests

At the end of the experiment, mice were fasted overnight and anesthetized with isoflurane, and blood was obtained from the eye sockets. These blood samples were centrifuged at 3,500 rpm for 15 min to collect serum samples, which were then immediately frozen at −80°C for biochemical assays. Lipid-related markers [triglycerides (TG), total cholesterol (TC), low-density lipoprotein (LDL-c), lactate dehydrogenase (LDH)] in serum were measured using an automated biochemistry instrument along with liver-function-related markers including alanine aminotransferase (ALT), alanoglutamic aminotransferase (AST), and alkaline phosphatase (ALP). Serum creatinine (CREA), serum urea (UREA) were also tested. Oxidative stress related indicators including glutathione peroxidase (GSH-PX), heme oxygenase 1 (HO-1) and oxidized malondialdehyde (MDA) levels were tested. Plasma indicator tests.

### 2.5 Enzyme-linked immunosorbent assay (ELISA)

ELISA kits (purchased from Huaying Institute of Biotechnology, Beijing, China) were used to detect the levels of inflammatory vesicles (NLRP3), IL-6 and C-reactive protein (CRP) in mouse serum.

### 2.6 Histopathological evaluation

Kidney tissue fixed with 4% paraformaldehyde was eluted with a gradient of water and ethanol, de-ethanolized with xylene, and embedded in paraffin. Paraffin blocks were sectioned, transparent with xylene, and then eluted with ethanol and water in a reverse gradient. Hematoxylin and eosin (H&E) staining, periodate-schiff (PAS) staining and Masson staining were used to assess the histopathological changes and degree of renal fibrosis.

### 2.7 Immunohistochemical

Changes in Nephrin expression observed by immunohistochemistry. Samples of tissue were embedded in paraffin, fixed in paraformaldehyde, and then sectioned into 4 μm thick pieces. After being dried at 60°C, the parts were cleaned with xylene to get rid of the wax. Following rehydration in graded alcohol, sections were incubated in 30% hydrogen peroxide. For the purpose of antigen repair, sections were incubated at 95°C in 10 mM citrate buffer (pH 6.0). The Nephrin (PRS2265, Sigma-Aldrich) antibody was then incubated on slides for a whole night at 4°C. The sections were also restained with hematoxylin after being stained with diaminobenzidine chromogen (ZSGB-BIO, China) and treated with secondary antibody for one hour at room temperature. A light microscope was used to view the stained slices.

### 2.8 Fasting blood glucose measurement

After 4 weeks of treatment, blood glucose levels were measured by tail vein using a glucometer (ACCU-CHEK, Germany), and the mice were fasted for 6 h before the measurement.

### 2.9 Western blot analysis

Total kidney protein was extracted using protein extraction reagent (ComWin Biotech, China). Equal amounts of proteins were demyelinated, separated by SDS-PAGE, and transferred to nitrocellulose membranes. After sealing, the membrane was incubated overnight at 4°C with primary antibodies, including CDK1 (abcam, United Kingdom), PAK1 (abcam, UK), Chk1 (abcam, United Kingdom), and β-actin (Proteintech). Subsequently, the membranes were washed again at room temperature and incubated with goat anti-rabbit antibody or rabbit anti-mouse antibody coupled with horseradish peroxidase for 1 h. The blots were then washed again and incu-blated using the Enhanced Chemiluminescent Protein Blotting Detection Kit (ComWin Biotech, China) followed by visualization of specific protein bands using eBlot.

### 2.10 Quantitative polymerase chain reaction assay

TRIzol reagent was used to isolate total RNA from cerebral ischemic infarction or colon tissue. RNA concentration was determined using a NanoDrop spectrophotometer (Thermo Fisher Scientific, United States). RNA was reverse transcribed to cDNA by using a reverse transcription kit (RR036A; TaKaRa Bio, Shiga, Japan). A fluorescence quantification kit was used (RR820A; TaKaRa Bio, Shiga, Japan). Relative quantification of mRNA expression was calculated using the 2^−ΔΔCT^ algorithm. Primers were as follows:

**Table:** 

Gene	Primer sequence (5′–3′)	
	Forward	Reverse
Cdk1	AGA​AGG​TAC​TTA​CGG​TGT​GT	GAG​AGA​TTT​CCC​GAA​TTG​CAG​T
CHK1	ACT​TAC​TGC​AAT​GCT​CGC​TG	TTG​AGG​GGT​TTG​TTG​TAC​CAT​C
p53	CCC​CTG​TCA​TCT​TTT​GTC​CCT	AGC​TGG​CAG​AAT​AGC​TTA​TTG​AG
PAK1	CAC​CAG​CAC​TAT​GAT​TGG​AC	ATT​CCC​GTA​AAC​TCC​CCT​GTG
FGFR1	TAA​TAC​CAC​CGA​CAA​GGA​AAT​GG	TGA​TGG​GAG​AGT​CCG​ATA​GAG​T
SIRT3	CTA​CAT​GCA​CGG​TCT​GTC​GAA	GCC​AAA​GCG​AAG​TCA​GCC​ATA
GR	AGC​TCC​CCC​TGG​TAG​AGA​C	GGT​GAA​GAC​GCA​GAA​ACC​TTG
ANGPTL4	CAT​CCT​GGG​ACG​AGA​TGA​ACT	TGA​CAA​GCG​TTA​CCA​CAG​GC

### 2.11 RNA-seq analysis

Standard extraction procedures were used to extract RNA from kidney tissues. Agilent 2100 bioanalyzer was primarily used for the rigorous quality control of the RNA samples, which allowed for the reliable detection of RNA integrity. To create the library, the 250–300 bp cDNA was screened using AMPure XP beads, followed by PCR amplification and further purification of the PCR products using AMPure XP beads. To find genes with significant variations in expression levels across states, gene expression was quantified and the expression data statistically analyzed. Additionally, GO function enrichment analysis and KEGG pathway enrichment analysis were carried out based on the functional roles of the genes.

### 2.12 Statistical analysis

GraphPad Prism 9.4 and SPSS 25.0 were used for statistical analysis. Data were presented as the mean ± SD. Multiple comparisons were performed using one-way ANOVA, followed by Tukey’s multiple comparisons test. The Kruskal-Wallis test was used for mNSS and immunofluorescence analysis. Statistical significance was set at *p* < 0.05.

## 3 Results

### 3.1 FGF21 improves renal function and glomerular filtration in mice with diabetic nephropathy

**FIGURE 1:**
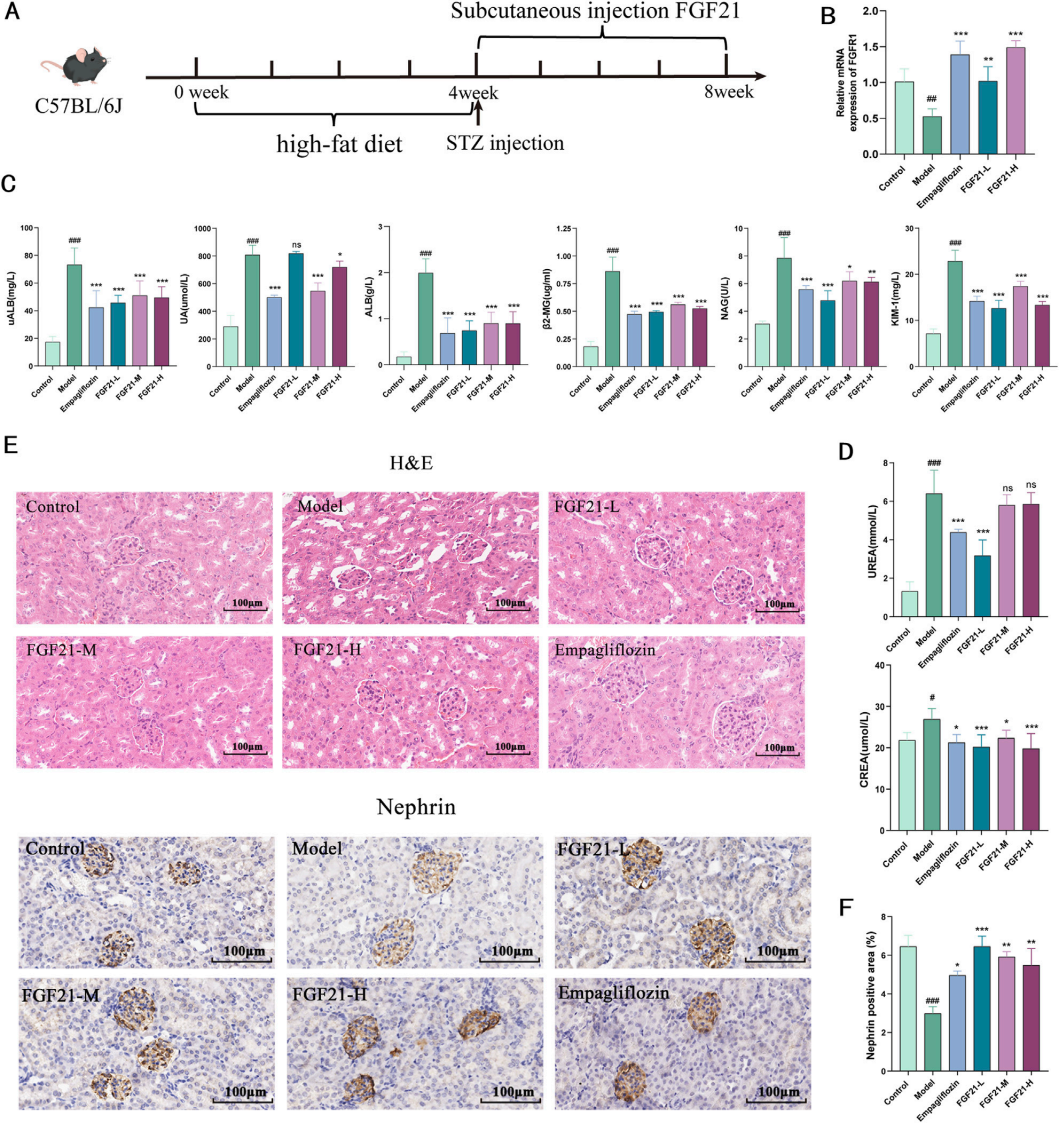
FGF21 protects glomerular filtration function and slows down renal injury in mice with diabetic nephropathy. **(A)** Diabetic nephropathy was induced in C57BL/6J mice by injection of STZ after high-fat 4, followed by subcutaneous injection of FGF21 for 4 weeks of treatment (n = 12 per group). **(B)** FGFR1 is a high-affinity receptor for FGF21 (n = 5 per group). **(C)** Urine was collected from mice after FGF21 treatment, and biochemistry instrumentation detected significant downregulation of urinary albumin (uALB), albumin (ALB) and uric acid (UA) levels, and decreased the levels of β2-microglobulin (β2-MG), and kidney injury factor-1 (KIM-1), and decreased the levels of N-acetyl-b-D-glucosylglucomutase (NAG) (n = 12 per group). **(D)** Biochemistry testing of serum, FGF21 decreased serum creatinine (CREA) decreased serum urea (UREA) levels (n = 12 per group). **(E)** H&E staining showed that FGF21 slowed down the modeling-induced glomerular swelling, glomerular basement membrane thickening with dilatation of the tethered area. Immunohistochemistry shows that FGF21 increases nephrin expression in glomeruli (n = 12 per group). **(F)** Quantitative results of Nephrin immunohistochemistry (n = 3 per group). Scale bars:100 μm. Data are presented as mean ± standard deviation (SD). ^*^ *p* < 0.05, ^**^ *p* < 0.01, and ^***^ *p* < 0.001 versus the Model group; ^#^ *p* < 0.05, ^##^ *p* < 0.01, and ^###^ *p* < 0.001 vs. the control group.

Diabetic nephropathy was induced in C57BL/6J mice by injection of STZ after high-fat 4, followed by subcutaneous injection of FGF21 for 4 weeks of treatment (Figure 1A). We examined the level of FGFR1, a high-affinity key receptor of FGF21, which significantly increased the mRNA level of FGFR1 (Figure 1B). Urine levels of urinary albumin (uALB), albumin (ALB), and uric acid (UA) were dramatically upregulated following STZ modeling, but subcutaneous injection of FGF21 significantly decreased the levels of these indexes (Figure 1C). Impaired glomerular filtration barrier after STZ modeling. Furthermore, STZ led to markedly increased levels of kidney injury factor-1 (KIM-1) and β2-microglobulin (β2-MG), as well as N-acetyl-b-D-glucosaminoglycans (NAG); remarkably, FGF21 was able to repair this injury (Figure 1B). Nevertheless, following modeling, serum urea (UREA) and creatinine (CREA) considerably rose, and FGF21 intervention decreased the levels (Figure 1D). Histopathology was used to investigate the morphological changes in the kidney. The results of H&E staining revealed that, following modeling, glomerular enlargement, thickening of the glomerular basement membrane, and dilatation of the thylakoid region were seen (Figure 1E). Crucially, the glomerular structural abnormalities considerably improved following the treatment of FGF21. Nephrin is a structural protein of the podocyte cleavage membrane that is only expressed in glomerular podocytes. Nephrin plays a vital function in maintaining the glomerular filtration barrier. Using immunohistochemistry, we were able to observe the expression of nephrin in the glomerular podocytes of mice in the model group, which was found to be significantly reduced (Figure 1F). In contrast, nephrin was significantly more expressed in the podocytes of mice in the administered group following the intervention of FGF21, which significantly improved the mice’s impaired glomerular filtration barrier caused by STZ.

### 3.2 FGF21 improves glycolipid metabolism in mice with diabetic nephropathy

**FIGURE 2:**
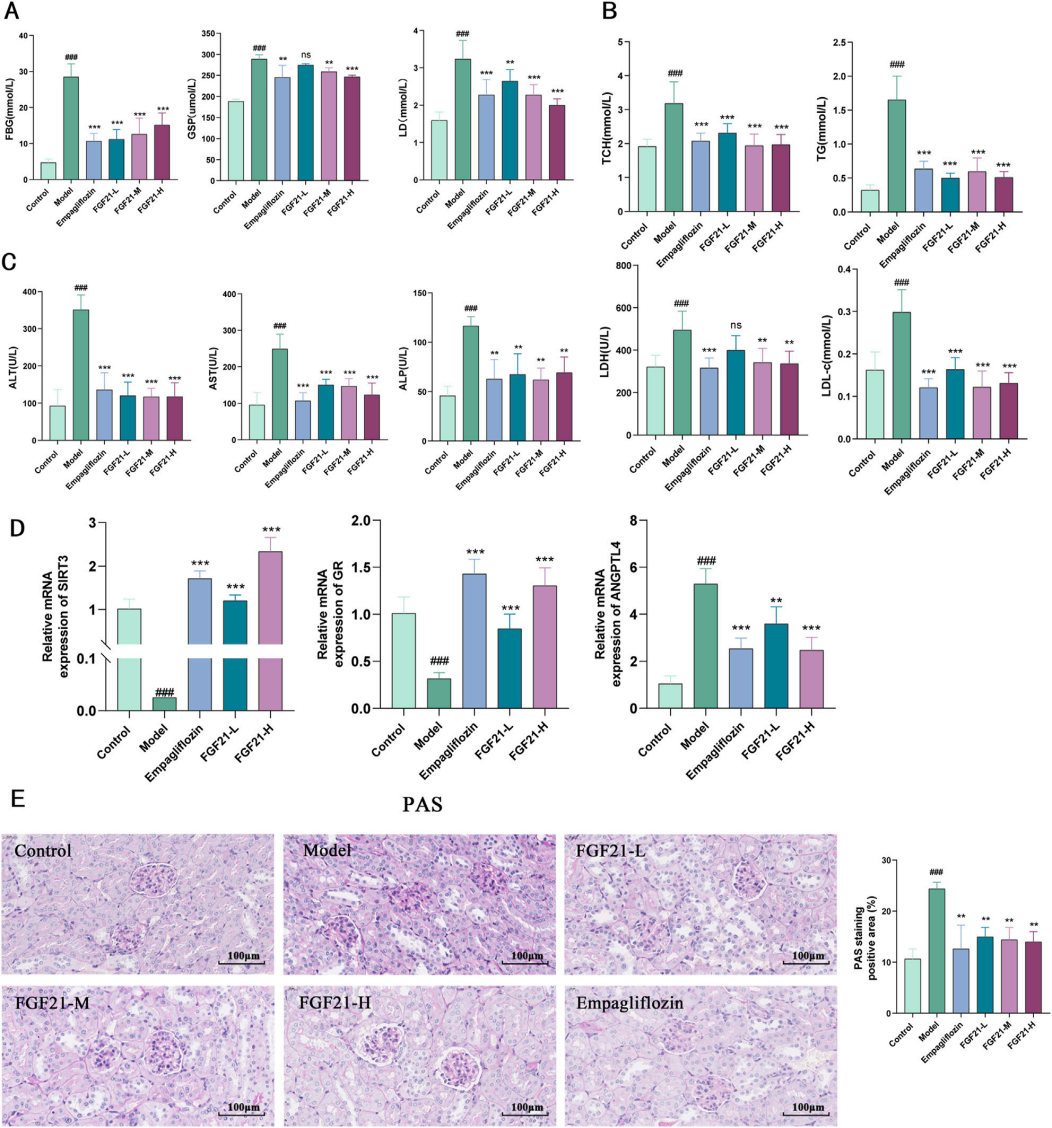
FGF21 slows diabetic nephropathy by improving disorders of glucolipid metabolism. **(A)** In mice, FGF21 lowers the levels of lactate (LD), glycated serum protein (GSP), and fasting blood glucose (FBG) (n = 12 per group). **(B)** Serum levels of lactate dehydrogenase (LDH), low-density lipoprotein (LDL-c), total cholesterol (TC), and triglycerides (TG) were all decreased by FGF21 (n = 12 per group). **(C)** Alkaline phosphatase (ALP), azelotransferase (AST), and alanine aminotransferase (ALT) were all found to be lowered in serum levels by FGF21 (n = 12 per group). **(D)** FGF21 elevated mRNA levels of Sirtuin3 (SIRT3) and glucocorticoid receptor (GR) and decreased mRNA levels of Angiopoietin Like 4 (ANGPTL4) compared to the model group (n = 5 per group). **(E)** PAS staining revealed that the amount of glomerular glycogen was decreased by FGF21 and quantification of PAS-stained positive areas is shown on the right side (n = 3 per group). Scale bars: 100 μm. Data are presented as mean ± standard deviation (SD). ^*^ *p* < 0.05, ^**^ *p* < 0.01, and ^***^ *p* < 0.001 versus the Model group; ^#^ *p* < 0.05, ^##^ *p* < 0.01, and ^###^ *p* < 0.001 vs. the control group.

Since disruption of glycolipid metabolism is the primary cause of diabetic nephropathy, we investigated the impact of FGF21 on this process. We found that STZ significantly increased the levels of lactate (LD), glycated serum protein (GSP), and fasting blood glucose (FBG) in mice (Figure 2A), but that FGF21 significantly attenuated the effect and reduced the disruption of glycolipid metabolism. On the other hand, FGF21 attenuated the influence of serum levels of triglycerides (TG) with total cholesterol (TC), low-density lipoprotein (LDL-c) with lactate dehydrogenase (LDH) in the mouse model group (Figure 2B). Since liver metabolic function has an important role in the development of diabetic nephropathy, we examined the changes of serum alanine aminotransferase (ALT), aspartate aminotransferase (AST), and alkaline phosphatase (ALP), and found that their levels were significantly increased after modeling, and this effect was suppressed by the intervention of FGF21 (Figure 2C). In addition we examined the levels of Sirtuin3 (SIRT3), a key protein for fatty acid oxidation, and glucocorticoid receptor (GR) and Angiopoietin Like 4 (ANGPTL4), key molecules for glycolipid metabolism. FGF21 was found to increase the mRNA levels of SIRT3 and GR, in addition to decreasing the mRNA levels of ANGPTL4 compared to the model group (Figure 2D). Meanwhile, we observed the effect of FGF21 on glycogen deposition in the kidneys by PAS staining, and we could see that after modeling, a large amount of glycogen deposition appeared in the glomerular thylakoid region, the glomerular basement membrane and around the tubules of mice, which was improved in FGF21-treated mice (Figure 2E).

### 3.3 FGF21 ameliorates oxidative stress and inflammation in mice with diabetic nephropathy

**FIGURE 3:**
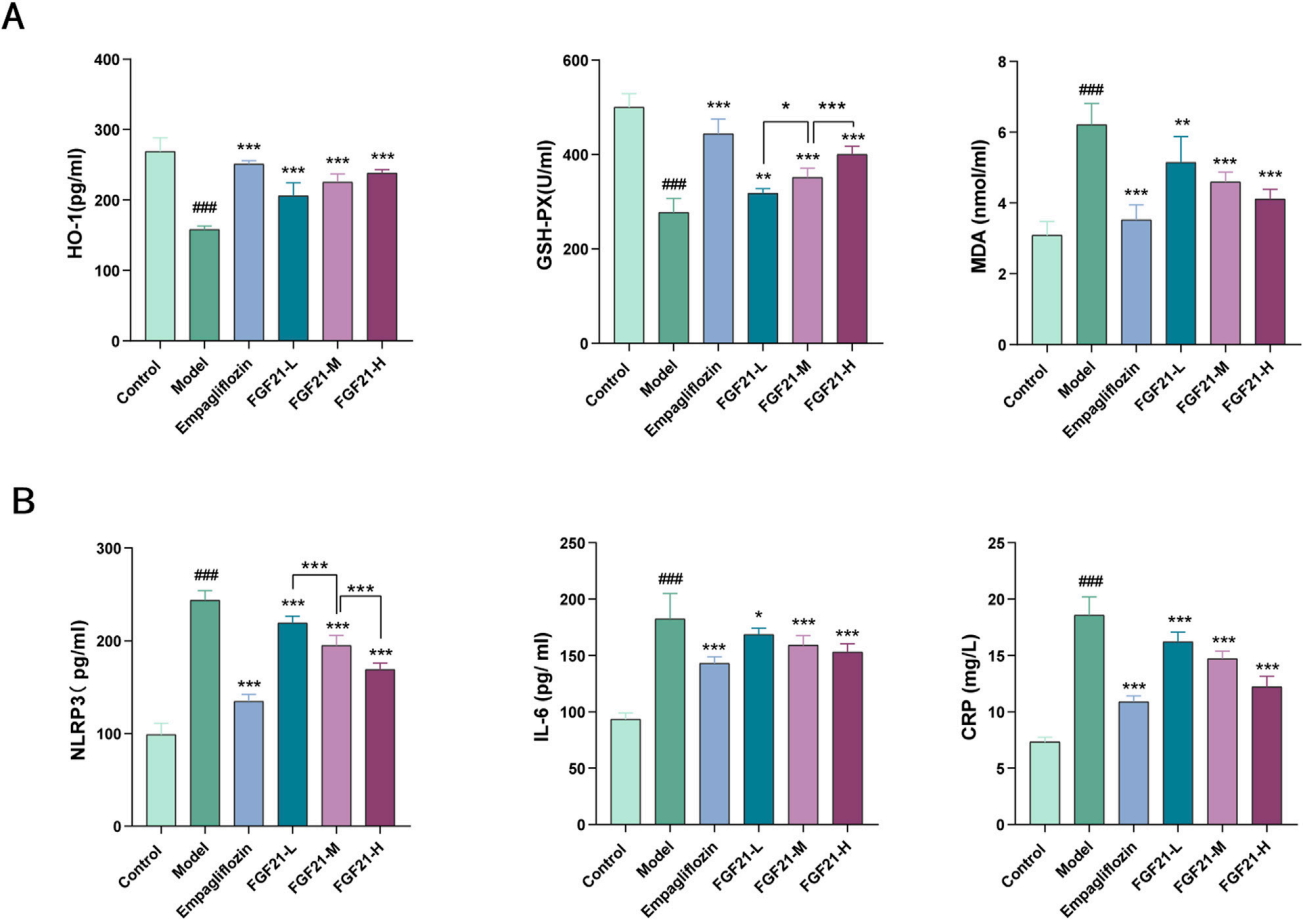
FGF21 attenuates oxidative stress, lipid peroxidation and inflammation ameliorates diabetic nephropathy. **(A)** FGF21 increased the expression of antioxidant enzymes [glutathione peroxidase (GSH-PX), heme oxygenase 1 (HO-1)] while decreasing the levels of oxidized malondialdehyde (MDA) (n = 12 per group). **(B)** FGF21 also significantly reduced the levels of inflammatory vesicles (NLRP3), IL-6 and C-reactive protein (CRP) (n = 12 per group). Data are presented as mean ± standard deviation (SD). ^*^ *p* < 0.05, ^**^ *p* < 0.01, and ^***^ *p* < 0.001 versus the Model group; ^#^ *p* < 0.05, ^##^ *p* < 0.01, and ^###^ *p* < 0.001 vs. the control group.

Hyperglycemia increases the *in vivo* glycosylation of antioxidant enzymes and reduces their activity, resulting in the build-up of free radicals that worsen kidney injury by increasing cytotoxicity, lipid peroxidation, and other related processes. As a result, we looked at the markers for oxidative stress and lipid peroxidation. After modeling, mice showed markedly elevated serum levels of inflammatory components and increased oxidative stress. When compared to the model group, FGF21 reduced the amount of oxidized malondialdehyde (MDA) and enhanced the production of antioxidant enzymes such as glutathione peroxidase (GSH-PX) and heme oxygenase 1 (HO-1) (Figure 3A). Furthermore, inflammatory vesicles (NLRP3), IL-6, and C-reactive protein (CRP) were all markedly decreased by FGF21 (Figure 3B). According to the information above, FGF21 was able to reduce inflammation and oxidative stress that accompanied the metabolism of glycolipids in diabetic nephropathy mice.

### 3.4 FGF21 can ameliorate the fibrinogen activation system in diabetic nephropathy mice, slow down fibrin deposition and improve renal fibrosis

**FIGURE 4:**
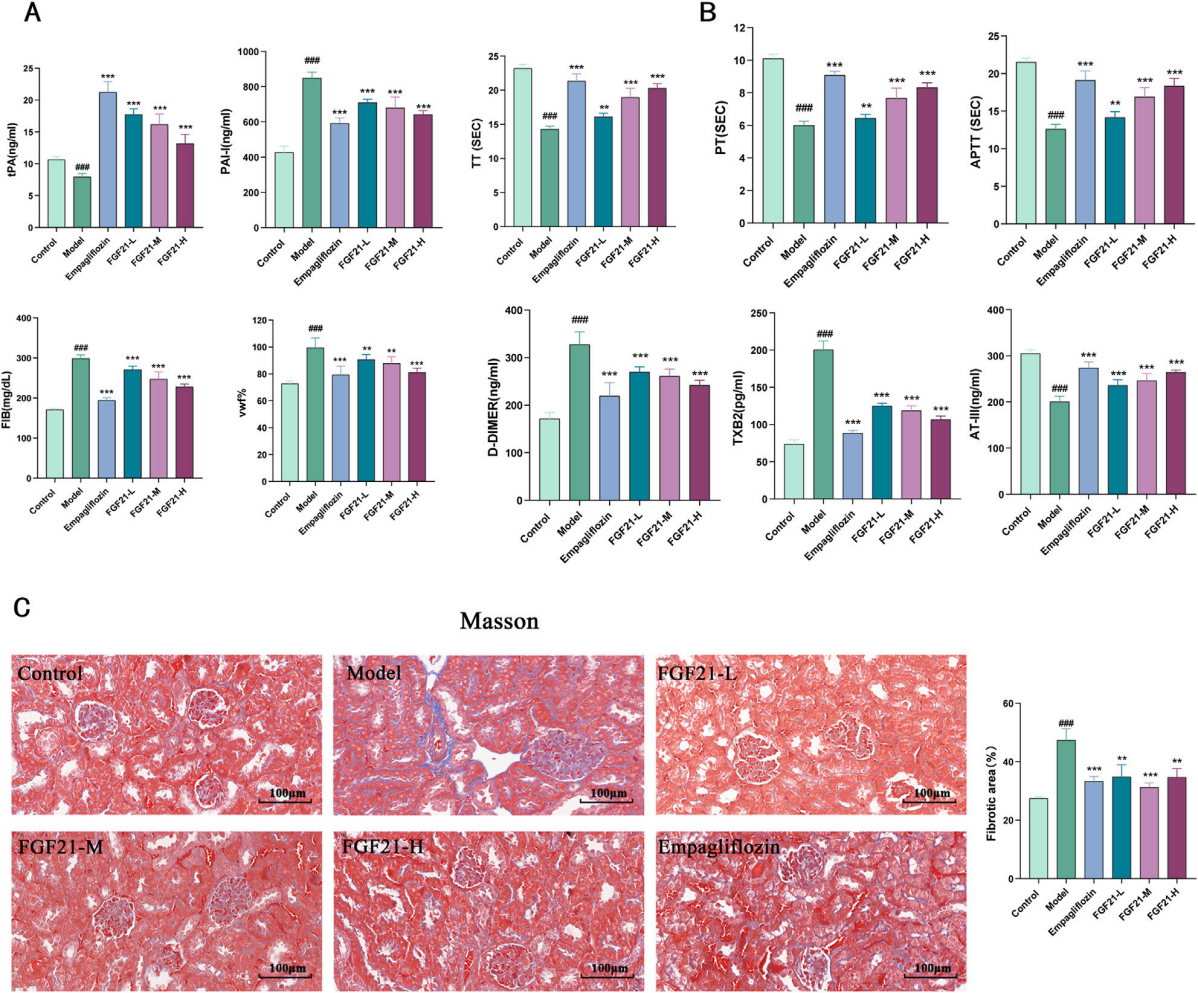
FGF21 protects renal function in mice with diabetic nephropathy by modulating renal hemodynamics. **(A)** FGF21 elevated plasma tissue plasminogen activator (tPA) levels and decreased fibrinogen activator inhibitor-1 (PAI-1), vascular hemophilic factor (VWF%), and plasma fibrinogen (FIB) levels (n = 12 per group). **(B)** FGF21 intervention prolonged prothrombin time (TT), activated partial thromboplastin time (APTT), and prothrombin time (PT), decreased thromboxane B2 (TXB2) and D-dimer (D-DIMER) levels, and increased antithrombin III (ATIII) levels (n = 12 per group). **(C)** Masson staining showed that FGF21 reduced collagenous fibrillar lesions appearing in glomeruli and Quantification of Masson stained fibrotic areas is shown on the right side (n = 3 per group). Scale bars: 100 μm. Data are presented as mean ± standard deviation (SD). ^*^ *p* < 0.05, ^**^ *p* < 0.01, and ^***^ *p* < 0.001 versus the Model group; ^#^ *p* < 0.05, ^##^ *p* < 0.01, and ^###^ *p* < 0.001 vs. the control group.

Patients with diabetes were shown to have reduced fibrinolytic activity, as demonstrated by aberrant tissue plasminogen activator (tPA) and plasminogen activator inhibitor-1 (PAI-1) activity. We therefore looked at the indexes related to plasma fibrinolytic activity and discovered that, following modeling, tPA was significantly reduced in mouse plasma, but levels of PAI-1, vascular hemophilic factor (VWF%), and plasma fibrinogen (FIB) were significantly elevated, which was reversed by FGF21 treatment (Figure 4A).

Among diabetic patients, vascular disease and injury are the leading causes of death. It was shown that following modeling, mice had lower plasma levels of anticoagulation-related substances, and that FGF21 intervention extended the duration of clotting (prolongation of prothrombin time (TT), activated partial thromboplastin time (APTT), and prothrombin time (PT). FGF21 also markedly raised antithrombin III (ATIII) and lowered thromboxane B2 (TXB2) and D-dimer (D-DIMER) levels (Figure 4B). Furthermore, we used Masson staining to observe the kidney’s fibrosis and noticed that STZ caused large collagenous fibrillar lesions in the glomeruli, which were ameliorated by FGF21 (Figure 4C). This indicates that FGF21 can alleviate renal fibrosis, slow down fibrin deposition, and activate the fibrinogen activation pathway in diabetic nephropathy mice.

### 3.5 FGF21 ameliorates diabetic nephropathy in db/db mice by reducing glomerular glycogen deposition and fibrosis

**FIGURE 5:**
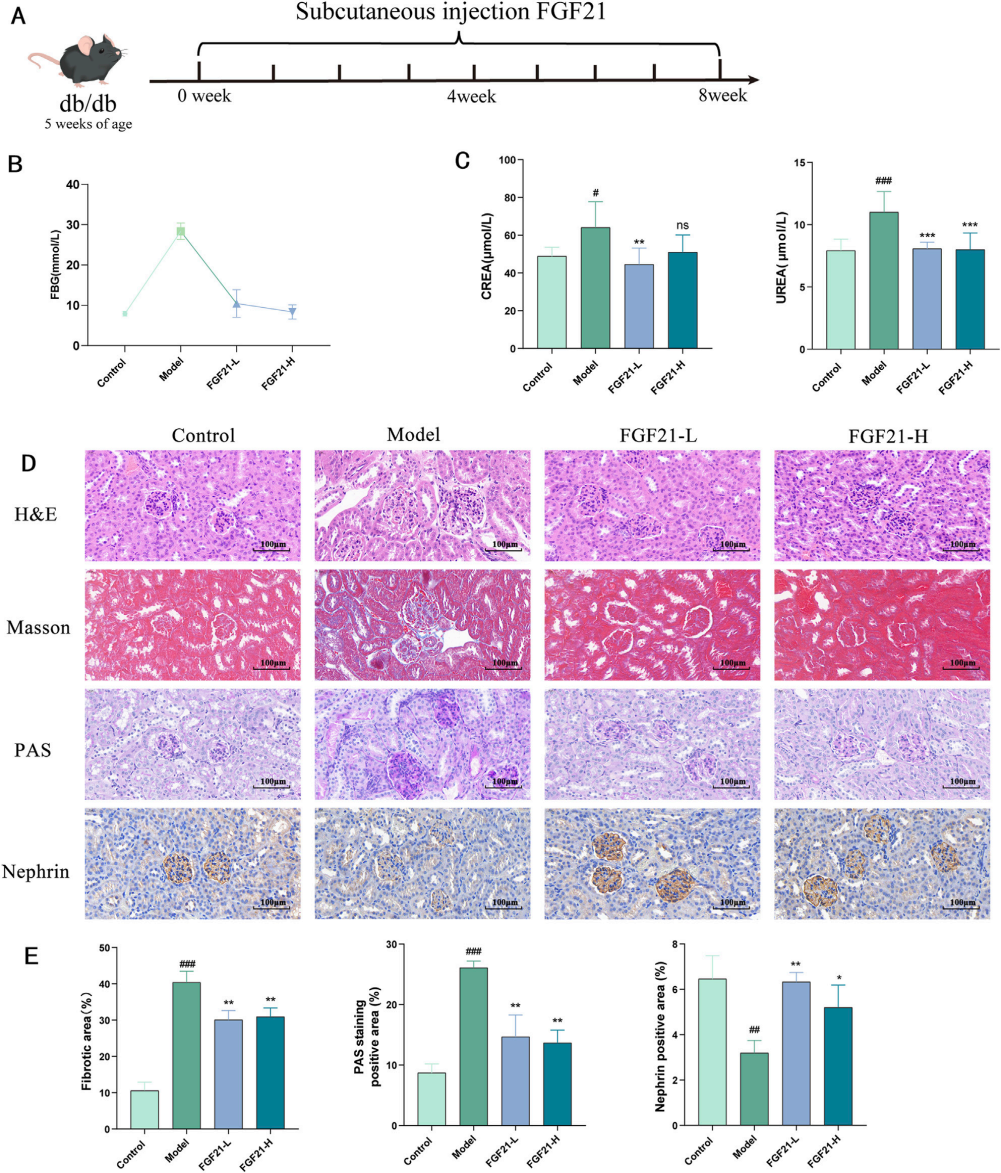
FGF21 ameliorates diabetic nephropathy in db/db mice by reducing glomerular glycogen deposition and fibrosis. **(A)** Pattern of FGF21 subcutaneous injection in 5-week-old mice for 8 weeks of treatment (n = 10 per group). **(B)** FGF21 significantly reduces fasting blood glucose in mice (n = 10 per group). **(C)** FGF21 could lower blood levels of urea (UREA) and creatinine (CERA) through biochemical testing on serum (n = 10 per group). **(D)** H&E results showed that FGF21 improved glomerular swelling and thickening of glomerular basement membrane. Masson staining showed that FGF21 intervention improved fibrosis. In addition, FGF21 reduced the amount of glomerular glycogen accumulation as shown by PAS staining. Furthermore, the results of immunohistochemistry demonstrated that FGF21 also raised the expression of nephrin. **(E)** Quantification of Masson stained fibrotic areas, PAS stained positive areas and nephrin immunohistochemistry positive areas (n = 3 per group). Scale bars: 100 μm. Data are presented as mean ± standard deviation (SD). ^*^ *p* < 0.05, ^**^ *p* < 0.01, and ^***^ *p* < 0.001 versus the Model group; ^#^ *p* < 0.05, ^##^ *p* < 0.01, and ^###^ *p* < 0.001 vs. the control group.

In order to corroborate the validity of the previously mentioned findings, we performed the pharmacodynamic analysis below using db/db mice, a type II diabetes model mouse caused by a mutation in the leptin receptor gene. Five-week-old db/db mice, acclimatized and fed for 1 week, were tested after 8 weeks of FGF21 treatment by subcutaneous injection (Figure 5A). We discovered that in db/db mice, FGF21 intervention was able to considerably lessen blood glucose levels (Figure 5B). Furthermore, we discovered that FGF21 could lower blood levels of urea (UREA) and creatinine (CERA) through biochemical testing on serum (Figure 5C). In addition we performed histopathological observations. H&E results showed that FGF21 improved glomerular swelling and thickening of glomerular basement membrane (Figures 5D, E). Results from masson staining indicated that FGF21 intervention improved fibrosis. Furthermore, as demonstrated by PAS staining results, FGF21 also decreased the amount of glomerular glycogen accumulation (Figures 5D, E). Additionally, we noticed that following FGF21 injection, there was an increase in the expression of the glomerular marker nephrin (Figures 5D, E). To sum up, FGF21 was effective in improving the defective glomerular filtration barrier and mitigating glycogen accumulation and fibrosis in the glomeruli of db/db mice.

### 3.6 FGF21 ameliorates diabetic nephropathy by mediating CDK1 regulation of the cell cycle

**FIGURE 6:**
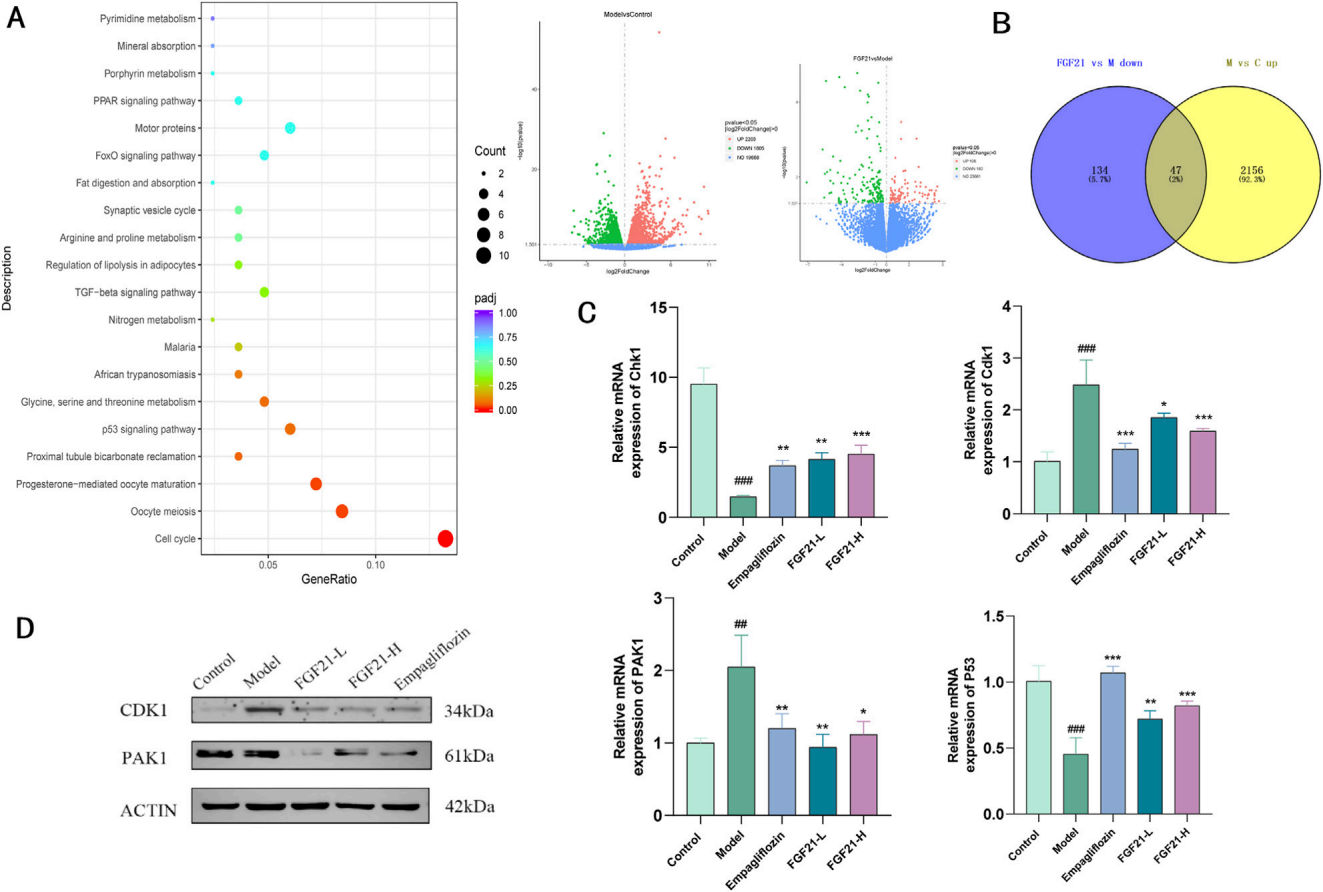
FGF21 regulation of cell growth cycle ameliorates diabetic nephropathy by modulating CDK1 expression. **(A)** KEGG analysis bubble plot results of genes significantly downregulated in the FGF21 administration group vs. the model group (left), the model group vs. the control group differential gene and the FGF21 administration group vs. the model group differential gene (right) (n = 4 per group). **(B)** Wayne plots of significantly upregulated genes in the model group vs. control group vs. significantly downregulated genes in the FGF21 administration group vs. model group (n = 4 per group). **(C)** FGF21 elevated cell cycle regulator-cell cycle protein-dependent kinase (CDK1) with P21 (RAC1) activated kinase 1 (PAK1) gene levels, while decreasing Checkpoint kinase 1 (Chk1) with P53 gene levels (n = 4 per group). **(D)** FGF21 reduced CDK1 and PAK1 protein levels (n = 4 per group). Data are presented as mean ± standard deviation (SD). ^*^ *p* < 0.05, ^**^ *p* < 0.01, and ^***^ *p* < 0.001 versus the Model group; ^#^ *p* < 0.05, ^##^ *p* < 0.01, and ^###^ *p* < 0.001 vs. the control group.

Transcriptome sequencing of kidney tissue from STZ-modeled C57BL/6J mice, we were able to determine the mechanism of action of FGF21. We discovered that 2203 genes were elevated in the model group when compared to the control group, and 182 genes were downregulated in the FGF21 intervention group when compared to the model group. KEGG analysis of these genes that were significantly downregulated in the FGF21 administration group relative to the model group showed that d large number of genes were enriched in the cell cycle and the differences were most significant (Figure 6A). In addition we obtained 47 genes by taking the intersection of genes downregulated in the FGF21 group vs. the model group and genes upregulated in the model group vs. the control group (Figure 6B). Among these genes, we discovered that cell cycle protein-dependent kinase (CDK1), to be a central molecule in the overall cell cycle regulatory mechanism. The results showed that FGF21 significantly reduced the expression of the CDK1 gene as well as upregulated the expression of its upstream factor Checkpoint kinase 1 (Chk1) (Figures 6C, D). In addition, it decreased the expression of P21(RAC1)-activated kinase 1 (PAK1) and increased the level of P53, a negatively regulated gene located upstream of CDK1 (Figures 6C, D). The above was supported by the Western blot analysis results. This indicates that via regulating CDK1 to regulate the cell cycle in renal tissues, FGF21 is likely to slow down the mitotic catastrophe and, consequently, the progression of diabetic nephropathy.

## 4 Discussion

**FIGURE 7:**
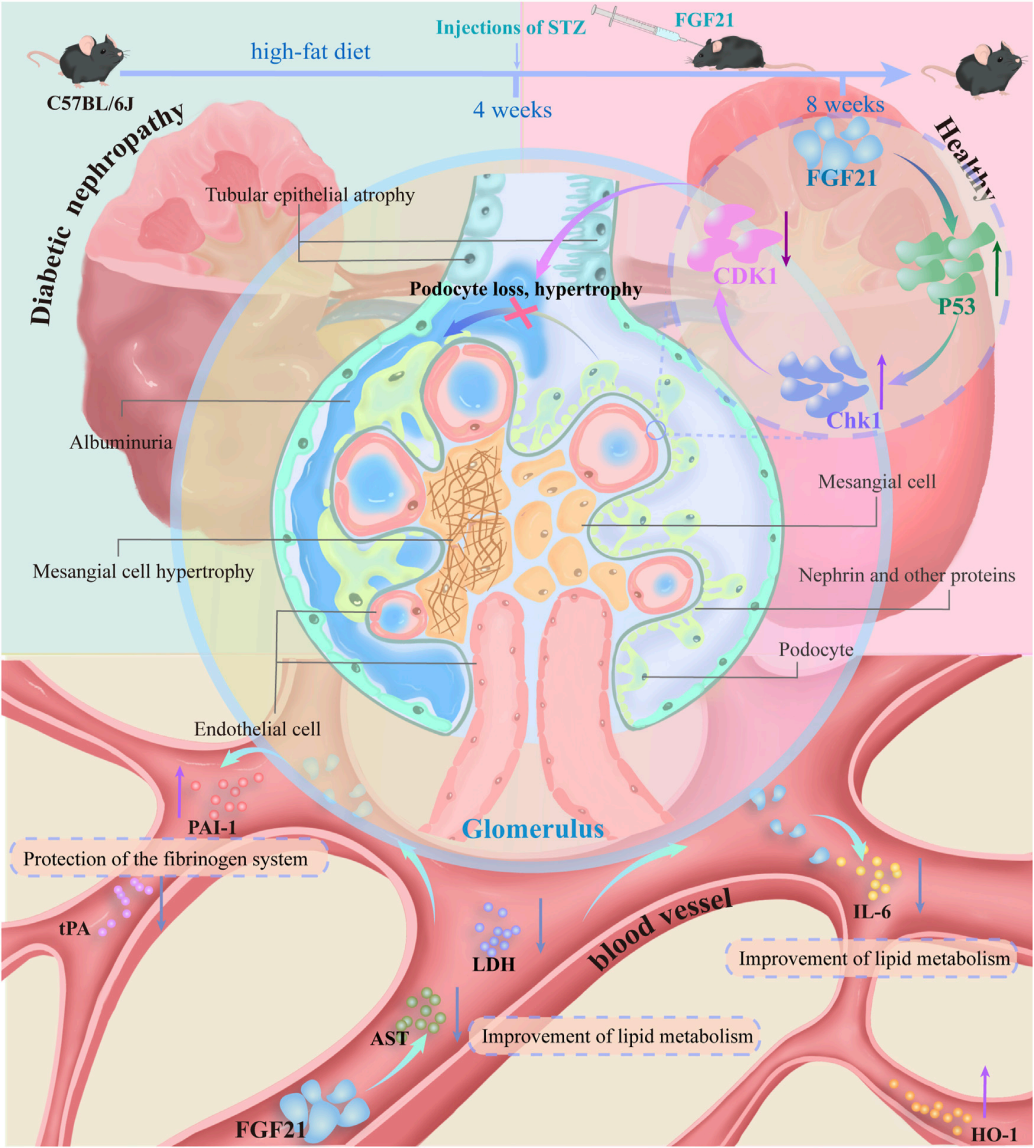
C57BL/6J mice on a high-fat diet for 4 weeks were injected with STZ resulting in mice becoming diabetic nephropathic mice. Mice with diabetic nephropathy will develop proteinuria, and the kidneys exhibit significant oxidative stress and inflammation with some fibrosis. In addition there may be concomitant vascular lesions. Glomerular podocytes are lost or swollen and the glomerular filtration barrier is impaired. This was followed by 4 weeks of treatment with subcutaneous injections of FGF21. FGF21 reduces the expression of the core cell cycle molecule CDK1 by decreasing the expression levels of elevated P53 and Chk1. This impedes the loss of podocytes, which in turn improves glycolipid metabolism (AST, LDH), inflammation (IL-6), and oxidative stress (HO-1), as well as the fibrinogen system (tPA, PAI-1) that accompanies diabetic nephropathy.

In this paper, using two models of diabetes, we demonstrated that FGF21 ameliorates proteinuria, oxidative stress and inflammation, as well as fibrosis and glomerular podocyte loss or swelling, and impaired glomerular filtration barrier in mice with diabetic nephropathy. In addition, transcriptomic analysis and experimental validation were performed on the kidneys of C57BL/6J mice. FGF21 was found to reduce the expression of the core cell cycle molecule CDK1 by decreasing the elevated levels of P53 and Chk1. This may impede the loss of podocytes, which in turn improves glycolipid metabolism (AST, LDH), inflammation (IL-6) and oxidative stress (HO-1), as well as the fibrinogen system (tPA, PAI-1) that accompanies diabetic nephropathy (Figure 7).

One of the main pathogenic processes of diabetic nephropathy is metabolic abnormalities; hyperglycemia changes the metabolism of endothelium and podocyte cells, overtaxing proximal tubular cells, a major source of cellular stress in the kidney (Mohandes et al., 2023; Audzeyenka et al., 2022). Hemodynamic changes are a result of diabetic nephropathy; further research has demonstrated that lipid metabolism abnormalities are a major factor in the onset and course of diabetic nephropathy. The buildup of free fatty acids in the diabetic kidney’s tubular epithelial cells causes lipotoxicity, which can result in inflammation, oxidative stress, and even cell death (Kim et al., 2018). FGF21 was found to improve glucose-lipid metabolism in this study by significantly lowering blood glucose levels, triglyceride and total cholesterol levels, and inhibiting blood glucose elevation (Figure 2B). We also observed decreased expression of inflammatory factors and attenuated oxidative stress (Figure 3). This shows that in the early stages of diabetic nephropathy, FGF21 may have a preventative and ameliorative effect.

The imbalance of the ADAMTS13-VWF axis due to chronic hyperglycemia may lead to thrombotic vascular disease in diabetic patients, while intrarenal thrombosis aggravates diabetic nephropathy (Rurali et al., 2013; Taniguchi et al., 2010; Dhanesha et al., 2017). Plasma tPA selectively activates fibrinogen to produce fibrinolytic enzymes, which can lead to the destruction of fibrin and thrombi to maintain blood flow to prevent ischemic injury and the accompanying inflammatory response (Thibord et al., 2021). Increased levels of PAI-1 in plasma diminish local fibrinolysis, decreasing the function of removing fibrin from blood vessels, and placing the organism in a hypercoagulable state, which results in an increase in blood viscosity increase, vascular stenosis and occlusion, insufficient perfusion, and small ischemic areas in the vessel wall, leading to secondary thrombosis (Kolseth et al., 2017; Le et al., 2008). In short, dysregulation of the dynamic balance between tPA and PAI-1 increases the risk of vascular and glomerulosclerosis and causes the blood to become hypercoagulable (Le et al., 2008). The current study found that FGF21 was able to significantly downregulate the level of PAI-1 in plasma while also raising the levels of the four coagulation components (PT, TT, APTT, and D-DIMER) (Figures 4A, B). This suggests that by promoting PAI-1 expression, FGF21 may be able to lower the risk of secondary thrombosis and delay the onset of diabetic nephropathy.

The development of glomerular disease into glomerulosclerosis and chronic kidney disease is largely dependent on the loss of foot cells. Podocytes are also unable to properly complete cytoplasmic divisions due to genetic, mechanical, immunologic, or toxic forms of triggers. This frequently results in the production of aneuploid podocytes, which rapidly separate and die, causing a mitotic catastrophe (Lasagni et al., 2010; Petermann et al., 2002; Neef et al., 2006). The podocyte undergoes a catastrophic mitotic event that damages it and results in podocyte loss, which compromises the glomerular filtration barrier and causes proteinuria (Brenner, 1985). Finally, terminally differentiated podocyte mitosis instead speeds up the loss of podocytes and glomerulosclerosis. Finding ways to enhance podocyte regeneration from other sources remains a challenging goal for improving chronic kidney disease treatment in the future (Lasagni et al., 2013). In the current investigation, we discovered that the mice experienced severe proteinuria, glomerular fibrosis, and podocyte loss in the model group. On the other hand, FGF21 treatment reduced proteinuria and enhanced the expression of the podocyte marker nephrin. We analyzed the mechanism of action of FGF21 through transcriptome analysis and performed KEGG signaling pathway enrichment analysis on genes that were significantly downregulated compared to the model group and found that cell cycle related genes accounted for the largest proportion and were highly significant. Taking the intersection of the genes downregulated by FGF21 relative to the model group and the genes upregulated by the model group and the control group, it was found that among these genes CDK1 is a core regulatory gene for cell cycle regulation, and further validated that FGF21 did significantly downregulate the expression of CDK1 gene and its protein (Figures 6A–C). CDK1 is a core protein in the regulation of the cell cycle G2/M, and inhibition of CDK1 will prevent cells from entering mitosis (Enomoto et al., 2009). It also inhibited the expression of PAK1 and P53, where PAK1 inhibition terminated mitosis and blocked cells in the G1 phase (Figure 6C) (Yi et al., 2023). It was found that CDK1 overexpression in adrenocortical carcinoma cell (ACC) lines locked ACC cells at the G2/M checkpoint by interacting with UBE2C and AURKA/B, promoting cancer cell proliferation and inducing epithelial mesenchymal transition (EMT) (Ren et al., 2022). In addition cellular G2/M arrest induces fibrosis (Wang et al., 2022). Importantly, it was found that premature activation of CDk1 can lead to mitotic catastrophe, which is an important factor in inducing podocyte loss (Castedo et al., 2002).

This indicates that in the treatment of diabetic nephropathy, CDK1 is anticipated to be a key target for controlling the cell cycle and reducing the loss of glomerular podocytes and associated fibrosis. Furthermore, by preserving the equilibrium of glycolipid metabolism, FGF21 was shown in this study to be able to further reduce the consequent oxidative stress, inflammation, and fibrosis. Moreover, by inhibiting the expression of CDK1, regulating the cell cycle, slowing down mitotic catastrophe, and reducing the loss of podocytes and eventually alleviating proteinuria caused by glomerular filtration barrier damage in diabetic nephropathic mice, FGF21 is expected to become a promising therapeutic drug to reduce podocyte loss and improve the prospect of chronic kidney disease.

## Data Availability

The original contributions presented in the study are publicly available. This data can be found here: https://www.ncbi.nlm.nih.gov/sra/?term=PRJNA1203032.
