# Supplementary material for: FGF21 ameliorates diabetic nephropathy through CDK1-dependently regulating the cell cycle
Source: Front Pharmacol. 2025 Jan 3;15:1500458. doi: 10.3389/fphar.2024.1500458 (PMC11739279; doi:10.3389/fphar.2024.1500458)
Supplement: Supplementary file 1 [file Image1.pdf]

Supplementary Material

1 Supplementary Figures

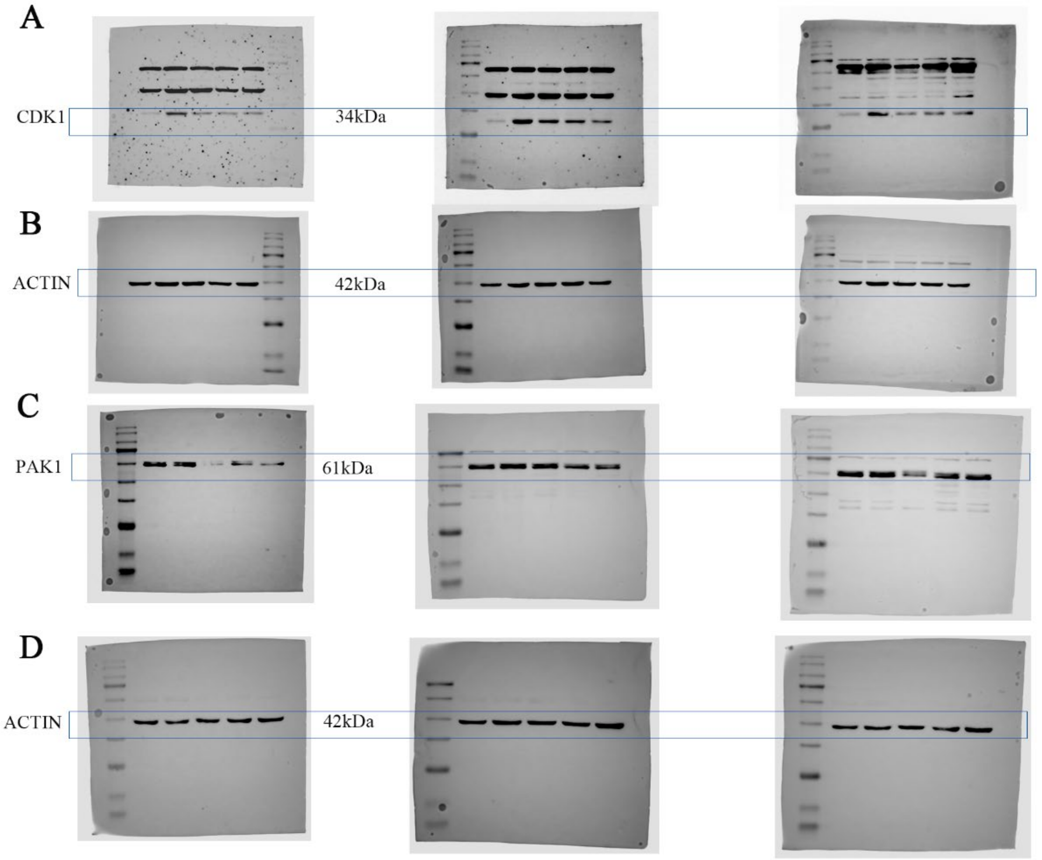

Western blot analysisy Raw data

Supplementary Figure 1. (A) Graph of the results of three replicate experiments for CDK1. (B) Graph of the results of ACTIN, the corresponding internal reference protein of CDK1. (C) Graph of the results of three replicate experiments for PAK1. (D) Graph of the results of ACTIN, an internal reference protein corresponding to PAK1.
